# Neural signal for counteracting pre-action bias in the centromedian thalamic nucleus

**DOI:** 10.3389/fnsys.2014.00003

**Published:** 2014-01-28

**Authors:** Takafumi Minamimoto, Yukiko Hori, Ko Yamanaka, Minoru Kimura

**Affiliations:** Department of Physiology, Kyoto Prefectural University of MedicineKyoto, Japan; Department of Molecular Neuroimaging, Molecular Imaging Center, National Institute of Radiological SciencesChiba, Japan; Brain Science Institute, Tamagawa UniversityMachida, Japan

**Keywords:** reward, thalamus, basal ganglia, attention, monkey, action-selection

## Abstract

Most of our daily actions are selected and executed involuntarily under familiar situations by the guidance of internal drives, such as motivation. The behavioral tendency or biasing towards one over others reflects the action-selection process in advance of action execution (i.e., pre-action bias). Facing unexpected situations, however, pre-action bias should be withdrawn and replaced by an alternative that is suitable for the situation (i.e., counteracting bias). To understand the neural mechanism for the counteracting process, we studied the neural activity of the thalamic centromedian (CM) nucleus in monkeys performing GO-NOGO task with asymmetrical or symmetrical reward conditions. The monkeys reacted to GO signal faster in large-reward condition, indicating behavioral bias toward large reward. In contrast, they responded slowly in small-reward condition, suggesting a conflict between internal drive and external demand. We found that neurons in the CM nucleus exhibited phasic burst discharges after GO and NOGO instructions especially when they were associated with small reward. The small-reward preference was positively correlated with the strength of behavioral bias toward large reward. The small-reward preference disappeared when only NOGO action was requested. The timing of activation predicted the timing of action opposed to bias. These results suggest that CM signals the discrepancy between internal pre-action bias and external demand, and mediates the counteracting process—resetting behavioral bias and leading to execution of opposing action.

## Introduction

In our daily life, most actions are selected and executed involuntarily, but they are appropriately incited by motivational, habitual or innate drive. For example, when actions are followed by different values of rewards, the highest one among the alternatives tends to be chosen frequently (Thorndike, 1898; Herrnstein, 1961), and to be executed quickly and accurately (Schultz et al., 1992; Watanabe et al., 2001; Minamimoto et al., 2005). Such a behavioral manifestation, the tendency or bias towards one over others (i.e., behavioral bias), reflects the consequence of action-selection or the decision-making process in advance of action execution. However, when we face unexpected situations (e.g., the highest option is unavailable), the pre-action bias is no more valid or even an obstacle, so that it should be withdrawn and replaced by an alternative that is suitable for the situation. This counteracting process is crucial to warranting our behavioral flexibility under unexpected situations, while pre-action bias allows us to execute actions efficiently without special effort. The two processes, internal-driven pre-action bias and external-driven counteracting to it, are considered to work in a complementary fashion.

Accumulating evidence suggests that the cortico-basal ganglia network, and especially the striatum, is a critical node for generating behavioral bias with respect to its role in action-selection or decision-making (Samejima et al., 2005; Hikosaka et al., 2006; Graybiel, 2008; Lau and Glimcher, 2008). In contrast, the neural basis for the counteracting process remains to be fully identified. A potential circuit is the thalamic centromedian-parafascicular (CM-PF) complex and its reciprocal connections with the cortico-basal ganglia system (Kimura et al., 2004; Minamimoto et al., 2009). Previously, we demonstrated that a subset of CM neurons of behaving monkeys responds to salient sensory stimuli (Matsumoto et al., 2001; Minamimoto and Kimura, 2002) and that it responds preferentially after instruction of actions associated with small reward while the behavioral bias toward large-reward action is manifested (Minamimoto et al., 2005). In addition, electrical stimulation of the CM nucleus mimics the counteracting process—slowing reaction to the larger-reward option (Minamimoto et al., 2005). These results suggested that CM plays important roles in detecting unexpected events and counteracting motivationally driven behavioral bias (Kimura et al., 2004; Minamimoto et al., 2009).

To understand the exact role of CM in the counteracting process, however, neural activity of CM needs to be better characterized in relation to behavioral bias in various situations. Here, we studied single-neuron activity in the thalamic CM nucleus while the monkey performed behavioral tasks with the following conditions: a GO-NOGO task in which two types of actions were associated with either large or small reward or were equally rewarded, and NOGO task in which only NOGO action was requested but with large or small reward instructed by visual signal. We found that CM neuron discharges after instruction for small-reward action signaled the discrepancy between the strength of pre-action bias and external demand to perform opposing action, the timing of which predicted the timing of opposing action. These results provide a better understanding of the role of CM in sensory-driven counteracting to internal pre-action bias.

## Materials and Methods

The present study was performed on the data that was partly published in a brief report (Minamimoto et al., 2005).

### Experimental animals

We used two male Japanese monkeys (*Macaca fuscata*): monkey SJ (5.8–7.5 kg) and monkey MA (6.7–8.0 kg). All surgical and experimental procedures were approved by the Animal Care and Use Committee of Kyoto Prefectural University of Medicine and were in accordance with the National Institutes of Health *Guide for the Care and Use of Laboratory Animals*. The monkeys had limited access to water for 4–5 days per week, but they received food and water *ad libitum* on weekends.

### Behavioral task

**Figure 1:**
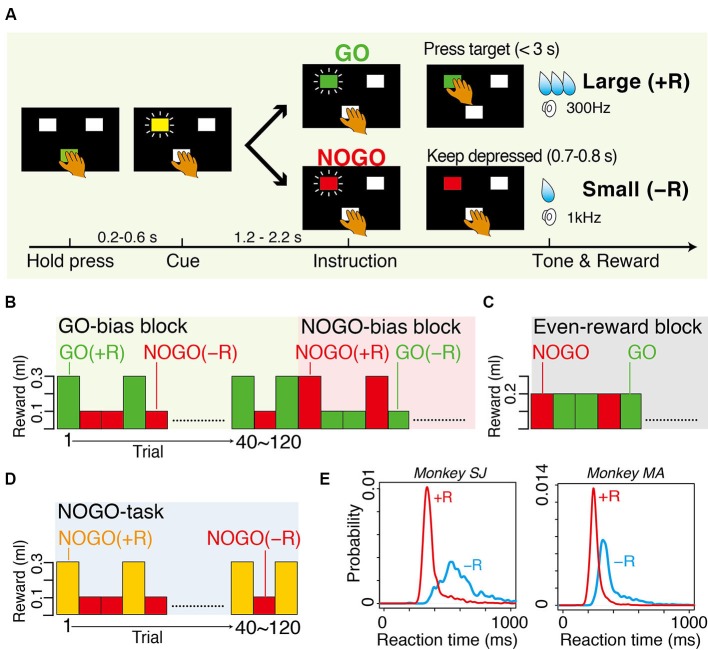
**Tasks and behavior. (A)** Sequence of events in GO-bias block of GO-NOGO task in which GO and NOGO actions were followed by large reward (+R) and small reward (−R), respectively. **(B)** Action-outcome associations in GO-bias and NOGO-bias blocks. GO (green) and NOGO (red) trials were asymmetrically rewarded in each block. **(C)** Action-outcome associations in even-reward block where reward size was equal in GO and NOGO trials. **(D)** Action-outcome associations in NOGO task in which only NOGO action was requested. Successful trials were rewarded with either large reward (+R) or small reward (−R), which was assigned by colored instruction (yellow or red). Timing of events was the same as that of GO-NOGO task. **(E)** Smoothed histograms of occurrence probability of reaction time. Red and blue curves are for GO(+R) and GO(−R) trials, respectively. Histograms have bin width of 1 ms and are smoothed with a Gaussian kernel (SD = 10 ms).

Monkeys sat in a primate chair in a sound-attenuated and electrically shielded room. They faced a panel in which a rectangular hold button and two instruction buttons were embedded. In the GO-NOGO task, when the monkeys pressed the hold button for 200–600 ms with their hand contralateral to the thalamus recording, one of two instruction buttons was illuminated yellow as a cue stimulus (Figure 1A). After an additional 1.2–2.2 s holding period, its color turned to either green or red, instructing GO or NOGO action, respectively. With the GO instruction, the monkeys had to release the hold button and press the illuminated target button within 3 s. With the NOGO instruction, the monkeys had to continue pressing the hold button for another 700–800 ms. In biased blocks, combinations of either a large water reward (0.3 ml, +R) after the successful GO trials and small water reward (0.1 ml, −R) after the successful NOGO trials or *vice versa* were run in single blocks of 40–120 correct trials (Figure 1B). In the even-reward block, successful trials were equally rewarded to both GO and NOGO trials (0.2 ml, Figure 1C). The occurrence of GO and NOGO trials was not predictable (average probability was 0.5). In NOGO task (Figure 1D), only NOGO actions were requested, but the reward, either a large (0.3 ml, +R) or small water reward (0.1 ml, −R), was given for successful trials. The reward size was indicated by colored instruction. A low (300 Hz) or high (1 kHz) tone was sounded after a correct behavioral reaction, which was followed by a large or small reward, respectively. For both GO-NOGO and NOGO tasks, when the monkey made an error, including failure to keep holding the button down and performing incorrect action, all LEDs flashed and the trial was aborted and the same trial condition was repeated. Through 1 month of training, the monkeys achieved performing the behavioral task at a high correct performance rate (>90%).

### Surgery

Surgery was performed under sterile conditions with the monkey under deep sodium pentobarbital anesthesia. Anesthesia was induced with ketamine hydrochloride (10 mg/kg, i.m.) and sodium pentobarbital (Nembutal; 27.5 mg/kg, i.p.), and supplemental Nembutal (6 mg/kg, i.m., for 2 h) was given as needed. Four head-restraining bolts and two recording chambers were implanted under stereotaxic guidance on the skulls of each monkey. The chamber for recording neuronal activity in the thalamus was positioned vertically over the thalamus. The center of the chamber was positioned midline and adjusted according to Horsley–Clark stereotaxic coordinates (anterior 12–13 mm). The other chamber was not used in this study.

### Electrophysiological recordings and data collection

We recorded the activity from single neurons that were located primarily in the CM nucleus as well as surrounding thalamic nuclei, such as parafascicular nucleus (PF) and dorsolateral PF (PFdl). Action potentials from single neurons were recorded using tungsten microelectrodes (2–5 MΩ at 1 kHz, FHC, Bowdoinham, ME) that were inserted through the implanted recording chamber and advanced by means of an oil-drive micromanipulator (MO-95; Narishige, Tokyo, Japan). The action potentials were amplified, filtered (50 Hz to 3 kHz) and isolated by spike sorter with a template-matching algorithm (multi-spike detector; Alpha Omega Technologies, Nazareth, Israel). Onset times of the action potentials were recorded on a laboratory computer (9821XV13; NEC, Tokyo, Japan) together with the onset and offset times of stimuli and the behavioral events such as pressing and releasing the button. In this study, we selectively studied the activity of long-latency-facilitation (LLF) type of neurons, which show burst discharges after unexpectedly presented auditory and/or visual stimuli of long latency (visual, 250–350 ms; auditory, 170–300 ms), such as knocks on the laboratory door. We also recorded licking movement by means of a strain gauge (DPM-711B; Kyowa, Tokyo, Japan) fixed to the waterspout.

### Data analysis

Analysis of behavioral and spike data and statistical test were performed using a Visual Basic (Microsoft, Redmond, WA) and R statistical computing environment (Team RDC, Vienna, Austria).

#### Behavioral data analysis

For behavioral data analysis, we excluded the data of the initial eight correct trials during the transitional phase between blocks of trials with different action-reward associations. Error rates for each trial type were calculated in each block, and were averaged across blocks in each bias condition. The average error rate for each trial type was compared between bias conditions by two-sample *t*-test. Reaction times (RTs, time between GO and releasing the hold button) and movement times (MTs, time between releasing the hold button and pressing the target) in GO trials were computed and compared between bias conditions by two-sample *t*-test.

#### Neural data analysis

For spike data analysis, we excluded the data of error trials and retrials after error trials, as well as eight successful trials after the block transition. Based on the previous study, we examined the discharge rates of each recorded neuron during two task epochs: (1) *Background*: the 250-ms period (500–750 ms) before pressing the hold button; and (2) *Post-instruction*: the 250-ms period (250–500 ms) after instruction onset. The statistical significance of changes in the discharge rate of the post-instruction activity for each of four trial types was evaluated by two-sample Wilcoxon test (*p* < 0.05) compared to the background activity. To quantify the preference of neural response, we performed receiver operation characteristic (ROC) analysis. For this analysis, we counted the number of spikes in the post-instruction period for each trial and constructed the distribution of spike numbers for each of GO(+R), GO(−R), NOGO(+R), and NOGO(−R) activity. Then we calculated the area under the curve of the receiver operating characteristic (ROC value) using a distribution set [e.g., GO(−R) and GO(+R)]. The ROC value gives us the general measure of selectivity; 0.5 indicates no preference while 0 and 1 indicate large- and small-reward preference, respectively. We examined the relationship between the latency of peak activation after GO instruction and RT in the same trial for each LLF neuron. First, we determined the peak activation after GO, although it was not detected in the remaining trials mostly because of the absence of spikes. To examine the relationship between neuronal activation and RT, we performed linear regression analysis on a trial-by-trial basis. For each trial, we determined the peak of activity (i.e., neural firing rate) smoothed with a Gaussian kernel (SD = 20 ms) during the period from the onset of GO instruction and 100 ms after GO reaction. Latency and magnitude of peak activity were used as regressors for multiple linear regression analysis of the GO RT.

### Identification of recording sites

At the end of all recording experiments, small electrolytic lesions were made at 8 and 16 locations along selected four and eight electrode tracks in monkeys SJ and MA, respectively. Direct anodal current (20 μA) was passed for 30 s through tungsten microelectrodes. After all studies were completed, the monkeys were deeply anesthetized with an overdose of sodium pentobarbital (Nembutal, 80 mg/kg, i.p.), and perfused with 4% paraformaldehyde. Half of the coronal 50-μm-thick sections were stained with cresyl violet (Nissl). For monkey SJ, the other half of the sections were stained by thiocholine method to demonstrate acetylcholinesterase (AChE) activity. The anatomical boarders of thalamic nuclei were assessed on histological sections by referencing the histological criteria of the monkey thalamus in conjunction with the assessment of their AChE activity. Histological reconstruction of the microelectrode tracks in relation to the electrolytic lesion marks allowed us to verify the location of the neuronal recordings.

## Results

### Behavioral bias and its counteraction

Two macaque monkeys performed in biased blocks of GO-NOGO task. Both average RTs and average MTs were significantly shorter in GO(+R) trials than in GO(−R) trials in both monkeys (RT, *p* < 0.001, *t*-test, Figure 1E; MT, *p* < 0.001, *t*-test). The monkeys made an error (either failure of GO reaction within 3 s or releasing the hold button in NOGO trials) more frequently in small-reward trials than in large-reward trials (GO, *p* < 0.01, in monkey SJ; NOGO, *p* < 0.01, both monkeys; *t*-test). These results suggest that, while large-reward action is facilitated by internal motivational drive, slowing of small-reward action is due to the conflict between internal bias and the external demand to overcome to it.

### Long-latency-facilitation (LLF) neurons preferentially respond to instruction of small-reward action

**Figure 2:**
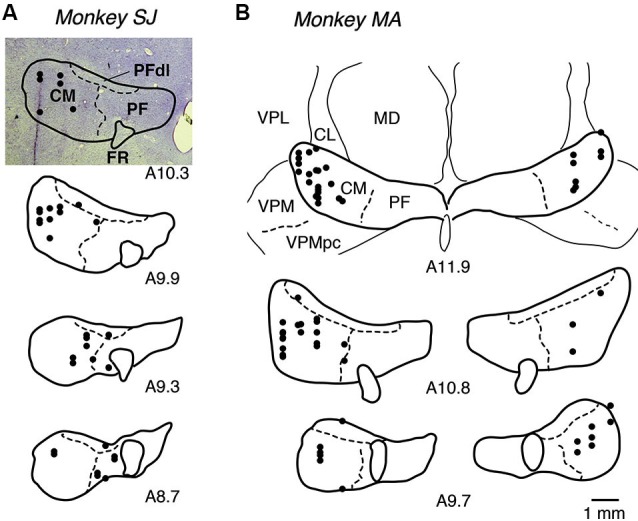
**Recording sites of LLF neurons. (A, B)** Locations of recording sites for monkeys SJ and MA, respectively. Locations of recorded neurons are plotted in black dots on photograph of coronal Nissl-stained sections (A10.3) or on drawings of borders of nucleus, positioned from anterior to posterior as from top to bottom. A10.3 represents anterior 10.3 mm in Horsley-Clarke coordinates (i.e., distance from the plane having external auditory meatus). CL, centrolateral nucleus; FR, fasciculus retroflexus; MD, mediodorsal nucleus; PF, parafascicular nucleus; PFdl, dorsolateral parafascicular nucleus; VPL, ventral posterolateral nucleus; VPM, ventral posteromedial nucleus; VPMpc, ventral posteromedial nucleus pars compacta.

We recorded the activity of 107 LLF–type neurons from the central thalamus (40 in monkey SJ and 67 in monkey MA) while the monkeys performed in a biased block of GO-NOGO task. LLF neurons were identified as showing burst discharges after unexpected auditory and visual stimuli with long latencies (Matsumoto et al., 2001; Minamimoto and Kimura, 2002; Minamimoto et al., 2005). We histologically confirmed that the locations of all 107 LLF neurons were in the thalamic CM nucleus and its vicinity, including the PF nucleus and PFdl (Figure 2).

**Figure 3:**
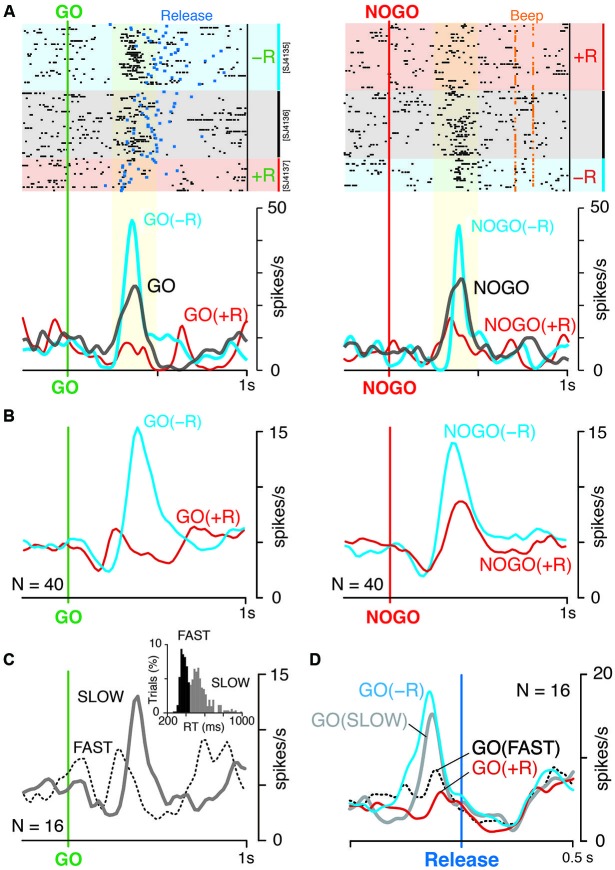
**LLF response to GO and NOGO instructions. (A)** Representative activity of LLF neuron responding to GO and NOGO instructions. Raster displays of spikes for NOGO-bias, even reward and GO-bias blocks are shown in order of occurrence of trials from top to bottom. Red, blue and gray shades indicate trials with large (+R, 0.3 ml), small (−R, 0.1 ml) and medium rewards (0.2 ml), respectively. Blue and orange marks in the raster plot indicate the time of hold-button release (Release; left) and the time of correct signal (Beep; right), respectively. Smoothed histograms (SD = 20 ms) for −R (blue) and +R trials (red) in biased blocks, and for trials in even-reward block (gray). Yellow shades indicate the time window of neural activity for quantitative analysis in Figure 4A. **(B)** Population histograms (smoothed, SD = 20 ms) of 40 LLF neurons in biased blocks. Activities are separately plotted by reward condition (+R, red; −R, blue). **(C)** Population histogram of 16 LLF neurons in even reward blocks. Activities are separately plotted by mode of RT as shown in inset (Fast, dotted curve; Slow, solid curve), in which bimodal distribution of RT in even-reward block are shown. Black and gray histograms assign the trials to fast (<440 ms) and slow mode (>440 ms), respectively. **(D)** Population histogram of 16 LLF neurons in even-reward blocks and that of the same neurons in GO-NOGO task. Colors assigned are the same as in **B** and **C**. All data shown were obtained from monkey SJ.

**Figure 4:**
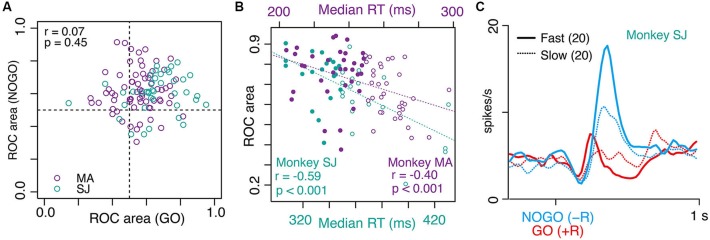
**Correlation between small-reward preference of LLF neurons and strength of behavioral bias. (A)** Scatter plot of small-reward preference of LLF neuronal responses to NOGO instructions (*y* axis) vs. GO instructions (*x* axis) measured by a window 250–500 ms after each instruction. Each data point corresponds to the ROC value derived from one neuron. The ROC value quantifies the separation of distributions for neural responses to −R and to +R (0.5 indicates no preference while 0 and 1 indicate perfect +R and −R preference, respectively). **(B)** Correlation between small-reward preference of each neuron (ROC value) and median RTs of GO trials in GO(+R)/NOGO(−R) block. Each data point indicates the ROC value of one neuron’s preference for NOGO(−R) relative to GO(+R) and median RT in the designated block. Filled and open circles indicate fast and slow half-blocks, respectively. **(C)** Population histogram (smoothed, SD = 20 ms) of LLF neurons (from monkey SJ) in GO(+R) (red) and NOGO(−R) (blue) trials in fast (solid line) and slow RT blocks (dotted line).

Figure 3A shows examples of the LLF response to GO and NOGO instructions. This LLF neuron showed phasic burst discharges after GO and NOGO instructions followed by small reward (−R trials; Figure 3A, blue shades and curves), whereas it showed almost no activation after instructions followed by large reward (+R trials; Figure 3A, red shades and curves). This was also evident in the population of activity; GO and NOGO responses of LLF neurons were higher in small-reward trials than in large-reward trials (Figure 3B). We quantified the reward preferences of GO and NOGO activity separately by using ROC analysis. Most recorded LLF neurons (78/107, 73%) showed small-reward preference for both GO and NOGO trials (ROC area > 0.5, Figure 4A). There was no significant correlation between small-reward preferences for GO and NOGO responses (Figure 4A, *r* = 0.07, *p* = 0.45). Collectively, these results indicate that LLF neurons preferentially respond to instruction for an action associated with a smaller reward.

### Small-reward preference positively correlates with strength of behavioral bias

In GO-NOGO task, the action-reward association was stable within a block of trials (GO(+R)/NOGO(−R) or NOGO(+R)/GO(−R); 40–120 trials), inducing a behavioral bias as shown above. However, even under the same action-reward association, the degree of behavioral bias varied block-by-block. For example, the median RT of GO(+R), an index of behavioral bias of a block, ranged from 307 to 430 ms, and from 204 to 297 ms, in monkeys SJ and MA, respectively. This gave us the opportunity to test whether the LLF preference of small reward is modulated by the strength of behavioral bias; assuming that recorded LLF neurons were sampled from a homogeneous population, the small-reward preference would be stronger when the neuron was recorded under stronger behavioral bias. To test this, we examined a block-by-block relationship between NOGO(−R) preference of the LLF response and the median RT of GO(+R) action. As shown in Figure 4B, there was a significant negative correlation between the neuronal preference for NOGO(−R) indexed by the ROC value and the median RT of GO(+R) trials in the designated block in which the neuron was recorded (monkey SJ, *r* = −0.59, *p* < 0.001; monkey MA, *r* = −0.40, *p* < 0.001; Figure 4B). When we split the population neurons in half according to the median RT in the block where the neuron was recorded, the NOGO(−R) response was much stronger in the fast-half blocks than in the slow-half blocks (Figure 4C, blue). However, the GO(+R) response did not differ between the two conditions (Figure 4C, red). Thus, when LLF neurons were recorded under high GO-bias, they tended to respond strongly to NOGO(−R) instruction.

We also examined the GO(−R)-NOGO(+R) block, where RT in GO(−R) trials was affected by the balance between pre-action bias and its counteracting. In this case, we could not find a consistent relationship; a significant negative correlation between GO(−R) preference and median RT was observed in monkey SJ (*r* = −0.54, *p* < 0.0001), but not in monkey MA (*r* = −0.16, *p* = 0.19).

### LLF activation related to counter-biased action without reward asymmetry

We examined the activity of 16 LLF neurons when a monkey performed in an even-reward block, with both GO and NOGO actions being equally rewarded (monkey SJ, Figure 1C). This condition without reward asymmetry resulted in a bimodal distribution of RT with an antimode at 440 ms (Figure 3C, inset), suggesting that the monkey internally generated behavioral bias to GO action in some trials and to NOGO action in others. The example LLF neuron responded to both GO and NOGO instructions; the response was stronger than that in large-reward trials but weaker than that in small-reward trials (Figure 3A, gray curve). To examine whether the LLF activity reflects internally generated behavioral bias without reward asymmetry, we divided all even-rewarded GO trials into two groups according to their RT, either faster or slower than the antimode (Figure 3C, inset). LLF neurons responded to GO instruction stronger in slow trials than in fast trials (Figure 3C). This was also evident in the population histograms for 16 LLF neurons aligned at the onset of the behavioral GO response as shown in Figure 3D. Prominent activation in slow GO trials in the even-reward block occurred with its peak preceded by about 130 ms to the onset of release (Figure 3D, gray curve). In contrast, activation in fast GO trials was not clear, but was seen with a small dip of the peak just before release (Figure 3D, dotted black). The contrasting activations and their time course in slow and fast trials in the even-reward block resembled those observed in GO(−R) and GO(+R) trials in biased block. Activities of all four conditions were indistinguishable at the onset of release and afterwards. These results suggest that, when behavioral bias is generated without reward asymmetry, LLF neurons discharge strongly before execution of counter-biased option, as observed when behavioral bias is induced by reward asymmetry.

### LLF response does not reflect small reward itself

**Figure 5:**
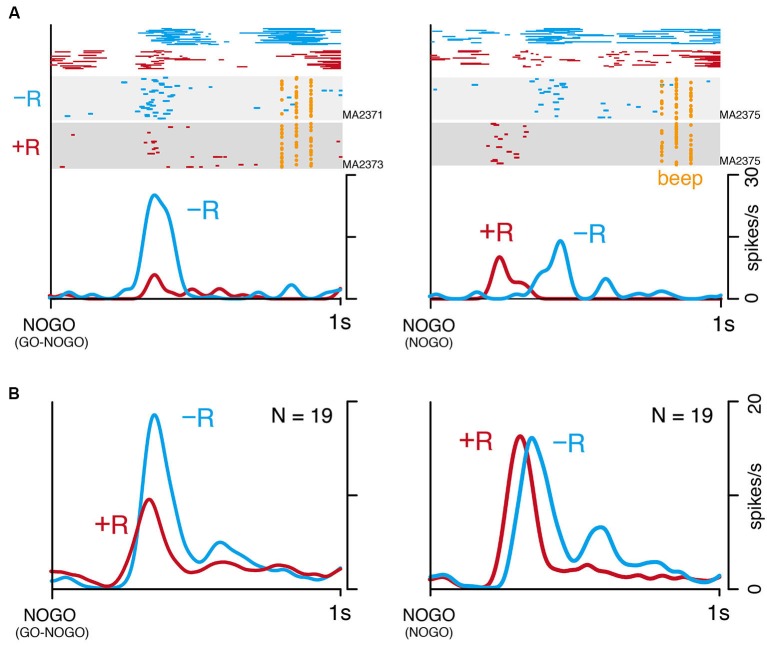
**Small-reward preference disappears when one action type is asymmetrically rewarded. (A)** An example of single LLF neuron response to NOGO instructions in GO-NOGO task (left) and NOGO task (right). Licking movement (top), raster (middle), and smoothed histogram (bottom) are separately plotted by reward condition (−R, blue; +R, red). **(B)** Population histogram (smoothed, SD = 20 ms) of 19 LLF neurons responding to NOGO instructions in GO-NOGO task (left) and NOGO task (right). Activities are separately plotted by reward condition (−R, blue; +R, red).

Although LLF activation after instruction for small-reward action seems to reflect behavioral bias as shown above, it could be a general signal related to small rewards. To examine this issue by dissociating small reward from counteracting process, we examined 19 LLF neurons in NOGO task (monkey MA, Figure 1D). In this task, the monkey was required to continue pressing the hold button in all trials, but it was informed by instruction that either large (+R) or small reward (−R) would be delivered. In GO-NOGO task, the monkey made stronger licking movements after NOGO(−R) instruction than after NOGO(+R) instruction (Figure 5A, top left). Similar patterns of licking were also observed in NOGO task (Figure 5A, top right), suggesting that the monkey recognized the rewarding condition by the instruction. An example LLF neuron showing strong response to NOGO(−R) in GO-NOGO task (Figure 5A, left) had similar discharge rates both after large- and small-reward instructions in NOGO task (Figure 5A, right). The population of 19 LLF neurons showed small-reward preference in GO-NOGO task (Figure 5B, left), but a similar discharge rate after two reward signals (Figure 5B, right), although timing of the activity was slightly different. These results suggest that the small-reward preference of LLF neuron activity does not reflect the general process regarding small reward.

### Timing of LLF activity explains well the timing of opposing action

**Figure 6:**
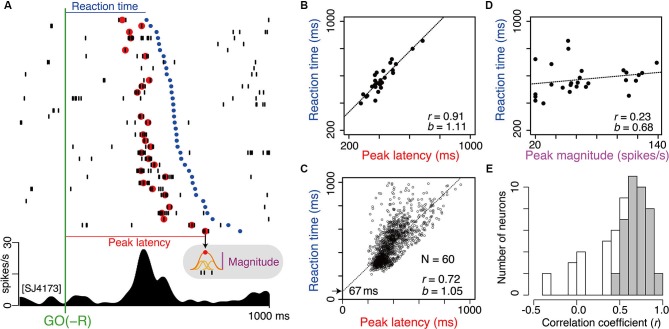
**Timing of GO(−R) activations well predict the timing of following GO(−R) action. (A)** Example of GO(−R) response aligned according to GO(−R) onset. Raster displays of spikes are shown in order of shorter RT from top to bottom. Red and blue dots indicate time of peak activity and onset time of GO(−R) action (release of hold button), respectively. Gray shaded inset indicates the schematic illustration of measuring the peak magnitude. **(B)** Relationship between peak latency of GO(−R) response and RT for the example neuron shown in **A**. **(C)** Same as **B** but for all significant GO(−R) responsive neurons (*n* = 60). **(D)** Relationship between peak magnitude of GO(−R) response and RT for the example neuron shown in **A**. **(E)** Histogram of correlation coefficient between peak latency and RT. Gray bars indicate neurons with significant correlation coefficient (*p* < 0.01).

As shown in Figure 3D, bias-dependent LLF activations occurred before onset of the action opposed to bias. To determine the specific process that LLF discharges would contribute to, it is important to understand the temporal relationship between LLF response and the following action. We analyzed the trial-by-trial relationship between the magnitude or timing of LLF activity after GO(−R) instruction and the timing of the following small-reward action (GO(−R)). For this analysis, we tried to detect the peak response for each trial. It usually originated from phasic burst discharge, which was a cluster of several spikes at a 3–10 ms interval. For example, in the neuron shown in Figure 6A, we detected peak GO(−R) activity (Figure 6A, red dots) in 27 of 36 (75%) trials. We performed this analysis on 60 LLF neurons that showed significant higher discharge rate in GO(−R) trials than baseline (*p* < 0.05, two-sample Wilcoxon test). We detected a peak response in average 68% of GO(−R) trials, and defined the magnitude and latency of the peak activity (Figure 6A; see Section Materials and Methods). Then, we performed multiple linear regression analysis of GO(−R) RT with peak latency and peak magnitude of GO(−R) response as regressors. There was a significant positive correlation (*p* < 0.01) between peak latency of GO(−R) activity and RT (Figure 6B). We found significant correlation in the majority (40/60) of neurons (Figure 6E, gray) as well as at the population level (*r* = 0.72, *p* < 10^-15^, Figure 6C). In addition, the regression line of the population (*b* = 1.05, intercept = 67 ms) indicated that the peak of LLF activity was constantly preceded to the following action (Figure 6C). In contrast, no neuron showed significant correlation (*p* < 0.01) between peak magnitude and RT (e.g., Figure 6D). This suggests that the timing of LLF activity for GO(−R) action can account for a trial-by-trial variance of RT among GO(−R) trials; the sooner LLF activity occurs, the sooner opposing action is executed.

We performed the same analysis on 35 LLF neurons that showed significant GO(+R) response in GO-bias block (*p* < 0.05, two-sample Wilcoxon test). We detected peak activity in relatively fewer trials (average 39%). We found significant correlation (*p* < 0.01) between peak latency of GO(+R) activity and RT less frequently (13/35, *p* < 0.05, χ^2^-test). In even-reward block, 9/16 neurons showed significant GO response. Peak latency was detected in an average 56% of trials. Significant correlation was found in 7 of 9 neurons. Together, the timing of LLF discharges can predict the timing of the following action, and especially action that has not been biased.

**Figure 7:**
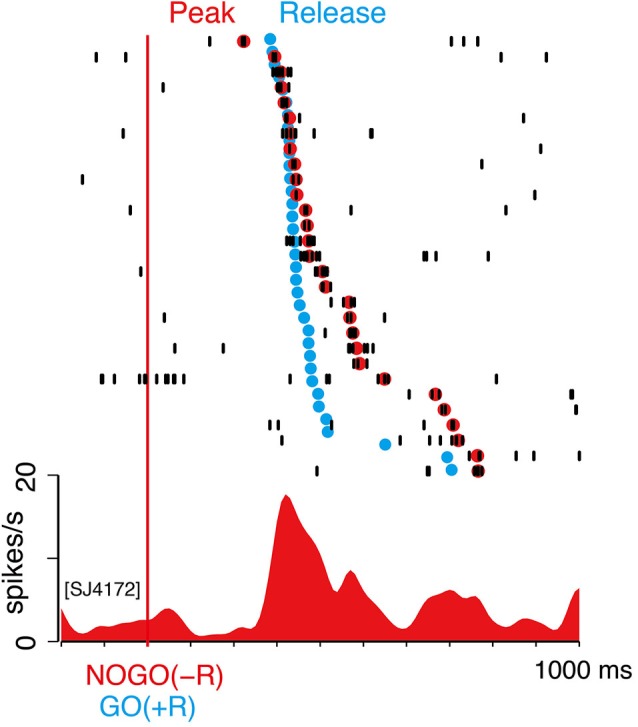
**NOGO(−R) activations did not precede GO(+R) action**. Example of NOGO(−R) response of the same neuron in Figure 6A; aligned according to NOGO(−R) onset. Raster displays of spikes are shown in order of shorter peak latency from top to bottom. Red dots indicate time of peak activity. The trials in which peak activity was not detected are not shown. Onset time of GO(+R) action in the same block is superimposed on the raster display by blue dots.

Similarly, we examined the timing of LLF activity after NOGO(−R) instruction in GO-bias block. In Figure 7, we marked the timing of peak discharge of the same neuron as in Figure 6A. Peak latency varied from 200 to 800 ms within a session (Figure 7), as with the case of GO(−R) response (Figure 6A). On the other hand, temporal variance of biased action (i.e., GO(+R)) was relatively small, as indicated by blue dots in Figure 7. Although a temporal comparison between peak NOGO(−R) response and GO(+R) reaction was not possible on a trial-by-trial basis, peak latency was relatively longer than the onset of GO(+R) in the same block (Figure 7). In 93 LLF neurons that showed significant NOGO(−R) response (*p* < 0.05, two-sample Wilcoxon test), median peak latency was significantly longer than the median RT of GO(+R) trials (*t*-test, *p* < 0.001). These results suggest that CM makes little or no contribution to the suppression of biased action.

## Discussion

In the present study, to investigate the neural mechanisms for counteracting pre-action bias, we tested monkeys performing GO-NOGO task, in which either GO or NOGO action was associated with large reward. The monkeys responded to instruction for large-reward action quickly and correctly, but reacted slowly to instruction for small-reward action. This suggests that, while large-reward action is facilitated by virtue of internal motivational drive (i.e., behavioral bias), slower small-reward action is due to a conflict between internal drive and external demand to overcome it (i.e., counteracting bias). LLF neurons, a subpopulation of neurons located mainly in the CM nucleus, exhibited phasic burst discharges after GO and NOGO instructions especially when associated with small reward. We found that the small-reward preference of the LLF response was positively correlated with the strength of behavioral bias toward large reward. A similar preference-bias relation was found in the block where both GO and NOGO actions were rewarded equally. When only one action type (i.e., NOGO) was requested with either large- or small-reward outcome, the small-reward preference disappeared. Furthermore, there was a positive temporal relation between LLF activation to GO(−R) instructions and the following GO(−R) actions on a trial-by-trial basis. LLF activations to NOGO(−R) instructions did not precede GO(+R) actions in the same block. Taken together, the results provide a better understanding of the role of CM in counteracting pre-action bias; CM neurons detect and signal external demand to overcome preset bias according to the degree of the bias.

As shown in a previous study (Minamimoto et al., 2005), most LLF neurons (>70%, Figure 4A) preferentially responded to instruction for small-reward action irrespective of action type. The preference for small-reward action was observed when actions associated with different magnitudes of reward. However, the differential activation of LLF neurons was also observed when two actions were equally rewarded; stronger activation occurred when instructions resulted in slow GO reaction trials compared to that in fast GO reaction trials in even-reward block (cf. Figure 3C). This suggests that the LLF response to instruction for an option is not a simple reflection of reward association in a categorical manner, but is also influenced by subjects’ internal bias. Indeed, under the same reward-action association, the response was affected by the degree of preset bias across the LLF population; as preset bias is strong, the response to the option opposed to bias also gets strong (cf. Figures 4B, C). This is consistent with the previous observation that the magnitude of the LLF response in a no-reward trial increases as expectancy of reward increases (Minamimoto et al., 2005). Together, these data suggest that neuronal response of CM reflects the discrepancy between internal preset bias and external demand for opposing action. Discrepancy signaling in the thalamic CM nucleus may be possible by integrating two sources of information from the basal ganglia and brainstem. The cortico-basal ganglia network has been implicated in a locus for creating pre-action bias (Hikosaka et al., 2006), and hence CM can gain access to pre-action bias by receiving axon collaterals of projections from the internal segment of the globus pallidus, the output nucleus of the basal ganglia, to the motor thalamic nuclei (Sidibe et al., 1997). In addition, CM receives projections from the brainstem pedunculo-pontine tegmental nucleus and the superior colliculus, both of which are considered to relay multi-modal aspects of sensory information (Pare et al., 1988; Grunwerg and Krauthamer, 1992; Krout et al., 2001). The thalamic CM nucleus thus appears to be located at an ideal position for coding discrepancy by monitoring pre-action bias and external events (Kimura et al., 2004; Minamimoto et al., 2009). Besides, CM may also receive discrepancy-related signal from the anterior cingulate cortex (Steriade et al., 1997; Hatanaka et al., 2003; Parent and Parent, 2005), which is suggested to play a role in conflict detection (Brown and Braver, 2005; Carter and Van Veen, 2007). Further studies are necessary to clarify how these inputs are integrated into discrepancy information and what the specific contribution of inputs from each brain structure to the integration is.

Discrepancy coding by CM neurons may raise the possibility that the CM contributes to the general process when a lesser reward than expected is assigned. One possibility is that the CM response might code the negative prediction error or negative motivational value, similarly to the neurons in lateral habenula (Matsumoto and Hikosaka, 2009). Another possibility is that the CM response may reflect disappointment or unpleasant process, since the CM-PF complex has been implicated in having a role in pain (Vogt and Sikes, 2000; Weigel and Krauss, 2004). However, those possibilities are inconsistent with our observation that LLF responses did not differ in magnitude between small- and large-reward trials when the same action was requested (in NOGO task, cf. Figure 5). It was also reported that LLF neurons similarly respond to salient stimuli irrespective of whether reward follows or not (Matsumoto et al., 2001). In contrast to magnitude, the latency of LLF activation was different between reward sizes in NOGO task (Figure 5). Although we do not have a good explanation for this result, it may not be a general property of LLF neurons since the latency difference was not found previously (Matsumoto et al., 2001). Collectively, our results suggest that CM does not have a general role regarding small reward.

Alternatively, discrepancy-related LLF discharges are likely to contribute to a specific process upon the request of opposing action. Discrepancy signaling by LLF discharges specifically occurred prior to the execution of opposing action regardless of with or without reward asymmetry (cf. GO(−R) and GO(SLOW) in Figure 3D). Although LLF neurons are activated by sensory stimuli even without motor response, phasic burst discharge after instruction for opposing action was not time-locked to the instruction, but temporally fluctuated trial-by-trial. Indeed, timing of the burst discharge predicted well the timing of the following GO(−R) action (cf. Figure 6). Given these action-related discharges, CM could have a direct role in the execution of opposing action. Still, this is unlikely because LLF neurons respond to instruction irrespective of action type. Given the above considerations, the most plausible interpretation for our results is that discrepancy-related LLF discharges mediate the counteracting process, which resets behavioral bias and leads to execution of opposing action.

Where does the counteracting process take place? The posterior putamen is a good candidate because it is the main target of the CM projections (Sadikot et al., 1992; Smith et al., 2004). Neurons in the striatum exhibit buildup activity toward an action instruction under asymmetrically rewarded condition (Lauwereyns et al., 2002; Takikawa et al., 2002; Hori et al., 2009), which is considered to be an underlying mechanism of creating advance bias for large-reward action (Hikosaka et al., 2006). The motivational bias is modulated by dopaminergic projections to the striatum (Schultz, 1998; Kawagoe et al., 2004). In the same GO-NOGO task, a subset of putamen neurons shows pre-movement activity specifically when one of two actions is associated with a large reward (Hori et al., 2009). When action opposing pre-action bias is unexpectedly requested, however, the striatal preset-bias-related activity becomes an obstacle to executing the requested action; the activity needs to be suppressed and/or overridden by opposing-action-related activity. In support of this, subsets of putamen neurons exhibit post-instruction activity according to specific, or combinations, of reward-action association(s), where the small-reward types were prominent and activated prior to onset of small-reward action (Hori et al., 2009). These counteracting processes can be triggered by the CM’s discrepancy signal transmitted through the thalamo-striatal projection. Concerning thalamic control of striatal activity, a potential substrate has been proposed by *in vitro* slice study (Ding et al., 2010). In brief, activation of thalamo-striatal axons induces burst activity in cholinergic interneurons, which leads to transient suppression of cortical input to medium spiny neurons (MSNs) and prolonged enhancement of responsiveness in striatopallidal MSNs. This suggests that thalamic burst activation can promote activity bias toward the “indirect-” over the “direct-pathway” of the cortico-basal ganglia circuit, which may lead to suppressing pre-action bias and unmasking opposing action. During GO-NOGO task, indeed, pre-GO action bias is diminished by electrical stimulation in CM, manifested as slower behavioral reactions in GO(+R) trials (Minamimoto et al., 2005).

In addition to the counteracting pre-action bias, CM burst discharges could also have a direct role in suppressing biased action. Although we could not test this hypothesis directly in a trial-by-trial manner, it is less likely because NOGO(−R) responses were not always ahead of time for initiation of GO(+R) action (cf. Figure 7). Instead, inhibition of the biased action may be accomplished by other brain systems, such as the subthalamic nucleus (STN), which is suggested to play a role in the inhibition of motor response (DeLong, 1990; Nambu et al., 2002; Isoda and Hikosaka, 2008).

As discussed above, our findings are consistent with the view that the CM-posterior putamen system complementarily operates between pre-action bias and counteracting it. This view can be extended to include PF and its connection with associative striatal regions (i.e., caudate nucleus and anterior putamen). Neurons in the caudate nucleus exhibit pre-movement activity that would create a motivational bias toward the contralateral space (Takikawa et al., 2002). On the other hand, neurons in the PF nucleus respond to salient sensory events especially when they appear in the contralateral location (Minamimoto and Kimura, 2002). Excitotoxic lesion or chemical inactivation of this nucleus impairs attentional orientating toward the contralateral hemifield (Mancia and Marini, 1995; Minamimoto and Kimura, 2002). Moreover, PF response to visual stimuli becomes stronger when it appears in unexpected places (Minamimoto and Kimura, 2002; Kimura et al., 2004). Thus, PF shares the same properties as CM in terms of counteracting internal bias, although it has not been tested in the context of motivational bias. Conversely, the contribution of CM may not be limited to counteraction to motivational bias. Indeed, when actions were equally rewarded, LLF discharges just before action depended on the strength of behavioral bias (cf. Figure 3D). As for eye-movement, Isoda and Hikosaka suggested that, while behavioral bias can originate from different domains (e.g., reflex, habit, motivational drive), the cortico-basal ganglia network is commonly involved in counteraction to it (Isoda and Hikosaka, 2011). In addition to the cortico-basal ganglia network, the counteracting process triggered by the CM-PF complex may also work for unexpected situations in general (Minamimoto et al., 2009). For example, when the subject unexpectedly detects salient stimuli or receives noxious stimuli, evoked CM-PF responses would contribute to resetting the on-going process in basal ganglia to facilitate impending behavioral reaction, such as attentional orienting or escape behavior. Future studies will have to investigate the significance of the CM-PF–striatal system in complementary operation of the counteraction to the pre-action bias originating from domains other than motivational drive.

Finally, our findings may also have a clinical significance, and especially for understanding cognitive deficits (e.g., set-shifting) in Parkinson’s disease (PD). Specific and remarkable (30–40%) neuronal loss in the CM-PF complex was demonstrated by postmortem brain studies in PD patients (Henderson et al., 2000a,b). The neuronal losses are selective to subpopulations of neurons: parvalbumin-positive neurons in PF and non-parvalbumin-positive neurons in CM (Henderson et al., 2000a). Anatomical tracing studies have shown that most of the CM neurons innervating the striatum are parvalbumin-containing (Sidibe and Smith, 1999), suggesting that CM-putamen projections are relatively intact in PD. Future study will have to identify the dysregulation of the CM-PF–striatal system caused by the degeneration of CM-PF in PD.

In summary, the present data demonstrated that neurons in the thalamic CM nucleus respond to external demand of action opposed to behavioral bias and signal the discrepancy between external demand and pre-action bias, the occurrence of which is followed by opposing action. The CM discrepancy signal may be used in its main target structure, the posterior putamen, to overcome its activity for the preset bias. This counteracting process seems to enable one to execute the opposite action, which is demanded externally but is not yet internally motivated or prepared. Interrelations between the basal ganglia and the thalamic CM-PF complex thus may allow us to switch our behavior properly and flexibly.

## Conflict of interest statement

The authors declare that the research was conducted in the absence of any commercial or financial relationships that could be construed as a potential conflict of interest.
